# Commentary: Winning a competition predicts dishonest behavior

**DOI:** 10.3389/fnins.2017.00417

**Published:** 2017-07-18

**Authors:** Yin Wu, Philip R. Blue, Luke Clark

**Affiliations:** ^1^Research Center for Brain Function and Psychological Science, Shenzhen University Shenzhen, China; ^2^Center for Brain and Cognitive Sciences and School of Psychological and Cognitive Sciences, Peking University Beijing, China; ^3^Department of Psychology, Centre for Gambling Research at UBC, University of British Columbia Vancouver, BC, Canada

**Keywords:** competition, testosterone, dishonesty, decision making, behavioral economics

Honesty is a crucial component in human cooperation, yet a large proportion of individuals choose to be dishonest on a daily basis (DePaulo et al., 1996). In fact, dishonesty is a pervasive problem that plagues society on both the global level (i.e., tax evasion leads to $3.1 trillion loss for governments annually; Tax Justice Network, 2011) and the local level (i.e., 32% of people are willing to lie on simple gumball tasks to increase their profit; Djawadi and Fahr, 2015).

Over the past several decades, researchers from economics (Gneezy, 2005; Charness and Dufwenberg, 2006), psychology (Mazar et al., 2008), and social neuroscience (Baumgartner et al., 2009; Zhu et al., 2014; Garrett et al., 2016) have been fascinated with understanding why people behave dishonestly. Classic economic theory suggests that people behave dishonestly when the benefits of lying outweigh the costs of getting caught (Becker, 1968), whereas an abundance of research suggests that certain psychological factors, such as perceived social class, can increase one's tendency to be dishonest (Piff et al., 2012). One proposed mechanism for this increase in dishonesty is a tendency for high-class individuals to be more oriented toward greed.

Recent findings by Schurr and Ritov (2016) demonstrated that winners were more likely to behave dishonestly following a social competition, either in a rigged laboratory task or even after recalling a past competitive victory. The effect was only reliable when the victory involved out-performing a competitor, and not when the win was determined by chance (on a lottery) or in reference to a personal goal (meeting a challenge). Schurr and Ritov's experiments ruled out the possibility that competence might explain their effects. The proposed mechanism, according to Schurr and Ritov, is that winning a competition increases feelings of entitlement, leading to subsequent dishonest behavior. Schurr and Ritov's experiments are well-designed and well-controlled, and we were impressed by their ingenious assay for dishonesty, based on the group deviation from chance on a dice-rolling game that allowed only the participant to cheat. However, their final experiment did not report the key test of mediation needed to support their hypothesis about entitlement. Indeed, their data provide no direct support for the role of entitlement in cheating after competitive wins.

The authors acknowledge that a variety of mechanisms could be at work, given the complex psychological nature of social competitions. We believe one strong candidate explanation, not acknowledged by Schurr and Ritov, is an increase in testosterone following competitive wins. Testosterone is one of the major sex steroids that plays a large role in human social interaction (see Eisenegger et al., 2011 for a review). The Biosocial Model of Status (BMS) posits that testosterone levels fluctuate as a function of competitive outcomes, such that winners show increased testosterone levels compared to losers (Booth et al., 1989). These endocrine changes have adaptive consequences for subsequent contests: wins enhance social status, and the testosterone rise facilitates competitive, aggressive behaviors needed to defend one's new position. For losers, the outcome signals a drop in social status, and a testosterone decrease may promote submissive behaviors that prevent physical harm or further loss of status. These effects were first observed in athletic competition and field data, and were further corroborated by using rigged laboratory tasks (Carré and Olmstead, 2015; Hamilton et al., 2015). Recent research suggests that testosterone promotes behaviors intended to maintain and seek social status rather than simply inducing aggressive behavior (Eisenegger et al., 2011). For example, testosterone potentiates aggressive responses to provocation, while it increases generosity in the absence of provocation in a modified Ultimatum Game (Dreher et al., 2016).

In Study 1, Schurr and Ritov adopted a conceptually similar rationale to the BMS, and the results were fully consistent with the predictions of the BMS. In Study 2, the authors replicated the effect by asking participants to recall an experience of winning a competition. Prior research has shown that the recall of a competitive victory elicited by watching a video clip of the win is sufficient to increase testosterone (Carré and Putnam, 2010). In Study 3, the critical role of social comparison was highlighted by showing that chance wins or achieving a personal goal did not induce dishonesty. Likewise, the outcome of a random lottery draw has no discernible influence on testosterone levels (Mazur and Lamb, 1980), and the tendency to attribute the success to external factors (i.e., luck and chance) buffered individuals' testosterone increases (Gonzalez-Bono et al., 1999, 2000). Given the role of testosterone in human social behavior, i.e., cheating (Lee et al., 2015), future experiments could fruitfully test the mediatory role of testosterone on dishonest behavior, which could help us to better understand the psychobiological precipitants of financial crises (Coates and Herbert, 2008) and have implications for future possibility of treating and/or preventing antisocial behavior.

## Author contributions

All authors listed have made a substantial, direct and intellectual contribution to the work, and approved it for publication.

### Conflict of interest statement

LC is the Director of the Centre for Gambling Research at UBC, which is supported by funding from the British Columbia Lottery Corporation and the Province of British Columbia. The other authors declare that the research was conducted in the absence of any commercial or financial relationships that could be construed as a potential conflict of interest.
